# Effects of Physical-Agent Pain Relief Modalities for Fibromyalgia Patients: A Systematic Review and Meta-Analysis of Randomized Controlled Trials

**DOI:** 10.1155/2018/2930632

**Published:** 2018-10-01

**Authors:** Yuichiro Honda, Junya Sakamoto, Yohei Hamaue, Hideki Kataoka, Yasutaka Kondo, Ryo Sasabe, Kyo Goto, Takuya Fukushima, Satoshi Oga, Ryo Sasaki, Natsumi Tanaka, Jiro Nakano, Minoru Okita

**Affiliations:** ^1^Department of Physical Therapy Science, Nagasaki University Graduate School of Biomedical Sciences, Sakamoto 1-7-1, Nagasaki 852-8520, Japan; ^2^Department of Rehabilitation, Nagasaki University Hospital, Sakamoto 1-7-1, Nagasaki 852-8520, Japan; ^3^Department of Physical Therapy, Niigata University of Health and Welfare, Shimami-cho 1398, Kitaku, Niigata 950-3198, Japan; ^4^Institute for Human Movement and Medical Sciences, Niigata University of Health and Welfare, Shimami-cho 1398, Kitaku, Niigata 950-3198, Japan; ^5^Department of Rehabilitation, Nagasaki Memorial Hospital, Fukahori 1-11-5, Nagasaki 851-0301, Japan; ^6^Department of Rehabilitation, Japanese Red Cross Nagasaki Atomic Bomb Hospital, Mori 3-15, Nagasaki 852-8104, Japan; ^7^Department of Rehabilitation, Saiseikai Nagasaki Hospital, Kitafuchi 2-5-1, Nagasaki 850-0003, Japan; ^8^Department of Rehabilitation, Juzenkai Hospital, Kago 7-18, Nagasaki 850-0905, Japan

## Abstract

**Purpose:**

We conducted a systematic review and meta-analysis to investigate the effects of the following physical-agent modalities for pain relief in fibromyalgia (FM) patients.

**Methods:**

We identified randomized controlled studies of adults with FM in the MEDLINE, CINAHL, and PEDro databases. The primary outcome measure was pain relief measured by a visual analogue scale (VAS), and the secondary outcome measures of interest were subjective improvements in the number of tender points, Fibromyalgia Impact Questionnaire (FIQ), and quality of life (QOL) scores.

**Results:**

Eleven studies were included in our review. The studies' physical-agent modalities were low-level laser therapy (LLLT), thermal therapy, electromagnetic field therapy, and transcutaneous electrical nerve stimulation (TENS). LLLT did not reduce VAS scores, but it significantly reduced both the number of tender points and FIQ score. Thermal therapy was associated with significantly reduced VAS scores, tender points, and FIQ scores. Electromagnetic field therapy was associated with significantly reduced VAS score and FIQ score. TENS significantly reduced VAS scores.

**Conclusion:**

Our analyses revealed that thermal therapy and LLLT had a partial effect on pain relief in FM patients, and this beneficial effect may have a positive influence on FM patients' health status.

## 1. Introduction

Fibromyalgia (FM) is an idiopathic, common, and complex syndrome, defined as long-lasting, widespread, and symmetrical nonarticular musculoskeletal pain with generalized tender points at specific anatomical sites [1, 2]. The pain that individuals with FM experience interferes with their performance of activities of daily life (ADLs) and results in a decreased quality of life (QOL) [2–5].

There are many possible treatments for FM that can be classified as pharmacological and nonpharmacological therapies [6–8]. The authors of a 2014 meta-analysis reported that very few drugs in well-designed clinical trials have demonstrated significant relief for multiple FM symptom domains, whereas nonpharmacologic treatments with weaker study designs have demonstrated multidimensional effects [8]. Nonpharmacological therapies such as physical exercise including strength training, aerobic training, and yoga [9, 10] and multicomponent therapy interventions [11, 12] have been used for FM. Physical-agent modalities are defined as passive treatments such as thermotherapy, cryotherapy, massage, electrotherapy, laser treatment, and others are nonpharmacological interventions used for FM patients [10]. Even though several placebo-controlled trials assessing the effects of physical-agent modalities on pain, ADLs, and QOL in patients with FM have been published in recent years, some studies had small sample sizes and have presented controversial results. A further elucidation of the effects of each physical-agent modality for FM is needed. We conducted the present study to systematically review the effects of physical-agent modalities for the treatment of FM, especially for the improvement of pain, ADLs, and QOL.

## 2. Methods

### 2.1. Search Strategy

We performed electronic searches of three databases—MEDLINE (the US National Library of Medicine bibliographic database), CINAHL (the Cumulative Index to Nursing and Allied Health Literature), and PEDro (the Physiotherapy Evidence Database)—up to February 28, 2017. A primary search with the term “fibromyalgia” was combined with the following terms: “cryotherapy,” “icing,” “low-level laser,” “laser therapy,” “electronical stimulation,” “TENS,” “electrotherapy,” “magnetic therapy,” “ultrasound,” “ultrasonic,” “thermotherapy,” “heat therapy,” “thermal therapy,” “shortwave,” “microwave,” “hot pack,” “wrapping,” and “traction,” and secondly, with “randomized controlled trial.” Reference lists of included articles were scanned for additional citations. The full search strategy is available upon request.

### 2.2. Study Criteria and Selection

Studies were included if (1) the participants were fibromyalgia patients; (2) the design was a randomized controlled trial (RCT) including crossover designs, published in peer-reviewed journals; (3) treatment using physical-agent modalities was compared with a pure control or placebo; and (4) the full text was available. Five independent reviewers screened the titles and abstracts of all retrieved citations for eligibility. Full-text articles were retrieved for review when they showed potential inclusion criteria or when there was insufficient information in the abstract and title to make a decision. Disagreements regarding selected articles were discussed between reviewers until a consensus was achieved, or a fifth reviewer was included to reach a majority decision. This systematic review is in accordance with the Preferred Reporting Items for Systematic Reviews and Meta-Analyses (PRISMA) statement.

### 2.3. Outcome Measures

The primary outcome measure was pain relief. The criterion that we used for the study's measurement of pain intensity was that the pain intensity had to be measured by a visual analogue scale (VAS) at the baseline and again after treatment. The secondary outcome measures of interest were subjective improvements in the number of tender points, the score on a Fibromyalgia Impact Questionnaire (FIQ), and the score for quality of life (QOL). The FIQ measures physical functioning, work status, depression, anxiety, sleep, pain, stiffness, fatigue, and well being. The studies' patients' QOL had to be measured by the Short Form 36 Health Survey (SF-36), Health Assessment Questionnaire (HAQ), or Arthritis Impact Measurement Scale (AIMS).

### 2.4. Data Extraction

The goal of our data extraction was to determine the differences between the studies' treatment groups regarding the mean outcome differences before and after treatment, and the standard error of these differences. The data were extracted independently by five investigators. The following data were extracted from each included study: participant demographics, the study design, the interventions, and the evaluation methods used for each group.

In studies in which multiple periods of treatment for pain were set, we analyzed the data for the longest period. In studies in which pain (as measured by a VAS) was treated in multiple body parts, the data for the part that had the strongest pain were analyzed. For the study's analysis, we required the mean difference between the baseline and the final data and the standard deviation of that difference for each group of subjects. When the required data were not described in studies, we calculated the mean difference and standard deviation using the study's data as described [13, 14]. Studies in which the required data could not be calculated were excluded.

### 2.5. Evaluation of the Studies' Methodological Quality

Two independent reviewers performed a quality assessment of each study by using the PEDro scale (Physiotherapy Evidence Database, 1999). This scale has shown good reliability for scoring RCTs [15]. The PEDro scale consists of 11 items related to scientific rigor. The scale's items 2 to 11 contribute to internal validity, and the study is given 1 point for each of these items that is met. The first item relates to external validity and is not included in the final score. The quality assessment was performed independently by the two reviewers, and any disagreement was discussed until consensus was reached.

### 2.6. Data Synthesis and Analyses

We performed the meta-analysis using Review Manager software, ver. 5.0 (Copenhagen, The Nordic Cochrane Centre, the Cochrane Collaboration, 2008) to determine whether the treatments using physical-agent modalities decreased the FM patients' pain. Outcomes were analyzed as continuous outcomes using a fixed-effect model to calculate the weighted mean difference and 95% confidence interval (95% CI). A *p* value ≤ 0.05 indicated significance for an overall effect. Heterogeneity was investigated using the chi-square test, and a *p*value ≤ 0.05 was accepted as significant. Subgroup analyses were also performed according to the physical-agent modalities.

## 3. Results

### 3.1. Database Search and Study Selection

**Figure 1:**
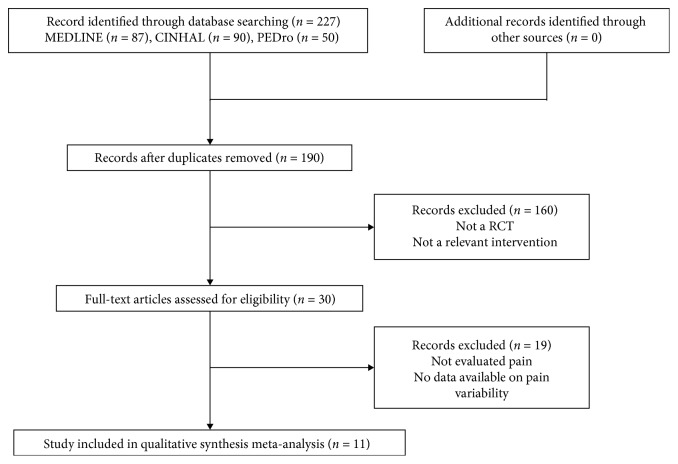
The different phases of the search of the three databases and the selection of the studies included in the present analyses.

Figure 1 illustrates the different stages of the search and the selection of studies included in our review. The initial search of the three electronic databases identified 227 titles and abstracts, of which 30 were retrieved for full-text review. When the exclusion criteria were applied, 11 studies satisfied the criteria to be included in this review [16–26]. The main reasons for exclusion were as follows: (1) outcomes of the pain scale were not reported or (2) the interventions and the comparison groups did not include a control group.

### 3.2. Quality Assessment of the Included Studies

A detailed description of the 11 studies' PEDro scores is shown in Table 1. Seven studies [17, 18, 21–23, 25, 26] showed a PEDro score >5, two studies [16, 17] scored 5, and the remaining two studies [19, 24] scored 4. The most frequent omissions in the studies were the lack of blinding of therapists (10 studies). The allocation of patients was not described in sufficient detail to ascertain whether the allocation was concealed in the randomization method (eight studies), and an “intention to treat” analysis was applied for least one key outcome (eight studies).

### 3.3. Characteristics of the Studies' Participants

The characteristics of the participants of the 11 studies are summarized in Table 2. The total number of participants was 498. Because two studies [23, 26] had a double treatment design, 28 subjects were excluded in this meta-analysis. The total number of participants included in the meta-analysis was thus 470. The treatment groups comprised a total of 236 FM patients and the control groups were a total of 234 participants. Detailed demographic data were not reported in all studies, but the majority of the participants were adults; one study [22] did not report the ages of the participants. The male: female ratio varied among the five studies [19–23], and the other six studies included only female participants [16–18, 24–26]. All participants (including the control group subjects) were patients with FM.

### 3.4. Characteristics of the Studies' Interventions and Physical-Agent Modalities

The interventions (i.e., the physical-agent modalities) applied in the 11 studies are summarized in Table 2. The most common intervention was low-level laser therapy, used in five studies [16, 21, 22, 24, 26]. Thermal therapy (which included balneotherapy, mudpack, and thermal bath) was used in four studies [16, 18–20]. TENS [23] and pulsed electromagnetic field therapy [25] were used in one study each.

The intervention protocols varied among the studies. The amplitude and irradiation density of the LLLT [16, 21, 22, 24, 26] were, respectively, applied to the tender point and the trigger point from 28 sec to 3 min. In thermal therapy [16, 18–20], the temperature ranged from 30°C to 45°C, and the adaptation time was from 10 to 30 min; several studies used 20 min. The TENS [23] was applied for 20 min, 2×/day for 7 days, and the intervention conditions were 200 *μ*sec, 2 and 100 Hz, and 60 mA. Pulsed electromagnetic field therapy [25] was applied for 30 min, 2×/day for 7 days, and the intervention condition was 40 *µ*T, 0.1–64 Hz.

### 3.5. Effects of Interventions

#### 3.5.1. Pain (as Measured by VAS)

**Figure 2:**
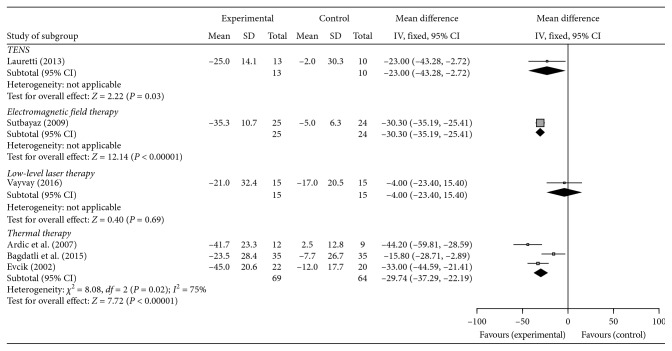
The mean difference and 95% CI of pain relief as measured using a VAS in 6 of the 11 studies for the physical-agent modalities: LLLT, thermal therapies, TENS, and electromagnetic field therapy.

Six of the 11 studies evaluated the participants' pain by means of a VAS and were included in the meta-analysis.  Figure 2 illustrates the mean difference and 95% CI values for pain relief as measured by VAS in these six studies for the physical-agent modalities LLLT, thermal therapies, TENS, and electromagnetic field therapy. The five studies' LLLT was not associated with the reduction of pain compared with the control group (mean difference: −4.00; 95% CI, −23.4 to 15.4, *p*=0.69). In contrast, the TENS (−23.00; 95% CI, −43.28 to −2.72, *p*=0.03), the electromagnetic field therapy (−30.30; 95% CI, −35.19 to −25.41, *p*<0.00001), and thermal therapy (−29.74; 95% CI, −37.29 to −22.19, *I*^2^ = 75%, *p*=0.02) were associated with a significant reduction of VAS score compared with the respective control group.

#### 3.5.2. The Number of Tender Points

**Figure 3:**
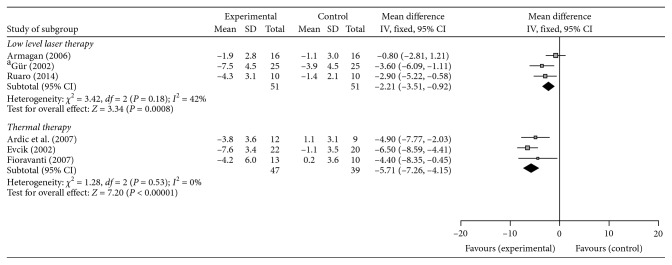
The mean difference and 95% confidence interval (CI) of tender points for physical-agent modalities.

Six studies evaluated pain by evaluating the number of tender points and were included in the meta-analysis. As illustrated in Figure 3, the LLLT (−2.21; 95% CI, −3.51 to −0.92, *I*^2^ = 42%, *p*=0.0008) and thermal therapy (−5.71; 95% CI, −7.26 to −4.51, *I*^2^ = 0%, *p*<0.00001) were both associated with a significant reduction of the number of tender points compared with the control group.

#### 3.5.3. The Fibromyalgia Impact Questionnaire (FIQ) Score

**Figure 4:**
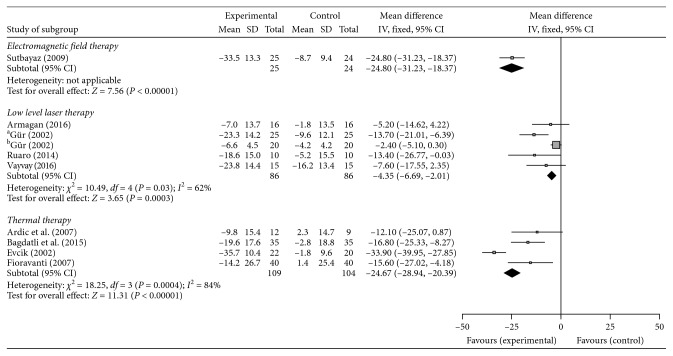
The mean difference and 95% CI values on the FIQ for the physical-agent modalities.

Ten studies evaluated the FIQ score and were included in the meta-analysis. As shown in Figure 4, electromagnetic field therapy (−24.80; 95% CI, −31.23 to −18.37, *p*<0.00001), LLLT (−4.35; 95% CI, −6.69 to −2.01, *I*^2^ = 62%, *p*=0.03), and thermal therapy (−24.67; 95% CI, −28.94 to −20.39, *I*^2^ = 84%, *p*=0.0004) were all associated with a significant reduction of FIQ score compared with the control group.

#### 3.5.4. Quality of Life (QOL)

Two studies evaluated the participants' QOL and were included in the meta-analysis. The LLLT as evaluated by SF-36 demonstrated no significant difference compared with the control group (5.80; 95% CI, −4.72 to 16.32, *p*=0.28). Thermal therapy, evaluated by HAQ and AIMS, demonstrated no significant difference compared with the control group (HAQ: −0.30; 95% CI, −0.93 to 0.33, *p*=0.35, AIMS: −0.40; 95% CI, −1.67 to 0.87, *p*=0.54).

## 4. Discussion

Fibromyalgia is defined as chronic pain, tenderness, and pain amplification [1, 27]. Increased levels of inflammatory cytokines and changes in neurotropic growth factors in the central nervous system and peripherally may influence the development and maintenance of central pain hypersensitivity by affecting adaptation and neuroplasticity [28–30]. The chronic painful lesions of fibromyalgia lead to limitations of activities of daily life and have been very difficult to treat effectively.

Fibromyalgia is characterized by a clinical syndrome whose primary symptoms include chronic widespread pain [1], and nonpharmacological options for fibromyalgia-induced pain may be as important as pharmacological treatment. Our meta-analysis revealed that TENS, electromagnetic therapy, and thermal therapy had positive effects on fibromyalgia-induced pain. These positive effects of nonpharmacological treatment may be due to physiological and biochemical changes in fibromyalgia patients. In two studies [23, 31], one of which was part of the present meta-analysis, the application of a TENS device improved pain relief in FM patients, and the effectiveness was suggested to be derived from a reduction in leukocyte migration, local action at peripheral opioids, and a decrease in local inflammatory reaction in the painful muscles. Low-frequency pulsed electromagnetic field therapy may improve pain in fibromyalgia patients, and several factors might mediate the therapeutic effects, such as alteration in pain perception, increases in the pain threshold and hormone levels, the inhibition of inflammatory edema, and vascular changes [25, 32]. Notably, only one RCT for TENS and one RCT for electromagnetic field therapy were identified. A further accumulation of RCT data regarding the effects of TENS and electromagnetic field therapy on fibromyalgia is needed. Ardic et al. [16] indicated that balneotherapy can effectively treat patients with fibromyalgia by relieving their clinical chronic pain, and they proposed that the suppression of inflammatory mediators with balneotherapy is related to its beneficial effect. Studies that examined hyperthermia showed that balneotherapy with mudpack and hot-pool treatments described a pain-relieving effect, which may be explained by a mitigation of muscle tone, increase in the pain threshold in the nerve endings, and/or peripheral vasodilatation [19, 20, 33].

Tender points were defined by the American College of Rheumatology criteria, which is the standard method for evaluating tenderness in fibromyalgia patients [34]. Our meta-analysis showed that the LLLT and thermal therapy were effective treatments for tenderness in fibromyalgia patients. That is, although Armagan et al. indicated that the numbers of tender points in their LLLT and placebo groups were not significantly different [17], the other two studies showed that the patients' tender point numbers decreased after LLLT [21, 24]. Our meta-analysis showed favors plot in LLLT group and that LLLT was thus an effective therapeutic method to reduce the number of tender points in FM patients. However, our meta-analysis indicated that LLLT did not effectively reduce the patients' VAS pain scores. On the other hand, three of the 11 studies in our meta-analysis that evaluated balneotherapy showed tender points' count was significantly different between the treatment group and nontreatment group, in addition to decrease in pain intensity [16, 19, 20]. This difference between physical-agent modalities may be derived from effective area of each modality. Many thermal therapies influence the body surface widely compared with the LLLT in the same therapeutic time; thermal therapy can treat multiple pain locations at a session. This has an advantage for treatment widespread pain of fibromyalgia patients. Therefore, thermal therapy reduces both pain intensity and the number of tender points. By contrast, LLLT may be ruled unfit to widespread pain of fibromyalgia patients because of narrow range of effective irradiated area. In the case of short-term treatment, LLLT may fail to decrease pain intensity in some painful areas, and then VAS in the patients remain persistently high. For this reason, LLLT may be more effective for decreasing the number of tender points than reducing the pain intensity. The therapeutic mechanism underlying LLLT remains to be elucidated in further studies.

The Fibromyalgia Impact Questionnaire (FIQ) comprised ten items in a self-administered instrument that measure physical functioning, work status, anxiety, pain, fatigue, sleep, depression, stiffness, well being, and evaluates activities of daily living (ADLs) in fibromyalgia patients [35]. EULAR guidelines emphasized that the goals of treatment are to improve the quality of life, maintain function (functional ability in everyday situations), and reduce symptoms [36]. In our meta-analysis, electromagnetic field therapy, LLLT, and thermal therapy were all associated with a significant reduction of the FIQ score. Three RCTs indicated that thermal therapy, including balneotherapy, mud baths, and mudpacks, had a positive effect on the FM patients' FIQ score, suggesting that thermal therapy ameliorated fibromyalgia-induced pain, and the improvement of fibromyalgia symptoms thus had a positive effect on the FIQ total score [10, 11, 13]. On the other hand, three of the five RCTs of LLLT reported that LLLT did not effectively reduce the FIQ score. Fibromyalgia-induced pain was not significantly changed in our meta-analysis, and this noneffectiveness may have led to the unchanged FIQ score. In addition, Bennett et al. suggested that a 14% change in the FIQ total score is clinically relevant [37]. In the results of LLLT, change in the FIQ total score is small compared with the clinically relevant value. Therefore, the positive effect of LLLT for fibromyalgia patients is smaller compared with thermal therapy and may be definite for the treatment of fibromyalgia.

Regarding the quality of the studies' evidence, although the PEDro score in nine studies was >5 (max. score = 9, min. score = 4), all nine studies were small-scale (the largest treatment group consisted of 40 participants). The intervention period in all nine studies was short (max. treatment period of 4 weeks). The quality of evidence according to Grades of Recommendation, Assessment, Development, and Evaluation (GRADE) for all outcomes of efficacy, tolerability, and safety was low, downgraded for the reasons given in the following description of study limitations.

The limitations are as follows. First, our review used only the MEDLINE, CINAHL, and PEDro databases for the search for studies, and we selected only English-language publications for the meta-analysis. We also selected only studies that included a pure control group or placebo groups (i.e., no other intervention). There are few reports on each physical-agent modality for fibromyalgia, and the heterogeneity analysis revealed a high score in the meta-analysis. Our meta-analysis did not evaluate the total effect of all of the physical-agent modalities since we searched for each modality's effect. Finally, the RCTs did not provide much data regarding the patients' QOL, and our search was thus unable to reveal adequate findings about posttreatment QOL in fibromyalgia patients. These restrictions are tasks to address in future studies.

## 5. Conclusions

In summary, our findings suggest that thermal therapy has a positive effect on fibromyalgia-induced pain, tender point, and FIQ. Thermal therapy is a more effective physical-agent modality for fibromyalgia patient treatment. Effect of electromagnetic therapy and TENS for the treatment of FM on pain intensity was observed. However, there are few reports on these physical-agent modalities. We speculate that this effectiveness has underlying mechanisms involving both the central nervous system and the peripheral nervous system. Clinically, nonpharmacological treatment for peripheral organization in fibromyalgia patients is important, and physicians need to consider both central and peripheral tissue as therapeutic targets.

## Figures and Tables

**Table 1:** Detailed description of PEDro scores.

Study (year published)	PEDro scores											
	1	2	3	4	5	6	7	8	9	10	11	Total of 2–11
Ardic et al. (2007) [16]	Yes	1	0	0	0	0	1	1	0	1	1	5
Armagan et al. (2006) [17]	Yes	1	0	1	1	0	1	1	0	1	1	7
Bagdatli et al. (2015) [18]	Yes	1	0	1	0	0	1	1	1	1	1	7
Evcik et al. (2002) [19]	No	1	0	1	0	0	0	0	0	1	1	4
Fioravanti et al. (2007) [20]	Yes	1	0	1	0	0	0	1	0	1	1	5
Gür et al. (2002) [21]	Yes	1	1	1	1	0	0	1	0	1	1	7
Gür et al. (2002) [22]	Yes	1	0	1	1	0	1	1	0	1	1	7
Lauretti et al. (2013) [23]	Yes	1	1	1	1	1	1	1	0	1	1	9
Ruaro et al. (2014) [24]	No	1	0	0	1	0	0	0	0	1	1	4
Sutbeyaz et al. (2009) [25]	Yes	1	1	1	1	0	1	0	1	1	1	8
Vayvay et al. (2016) [26]	Yes	1	0	1	0	0	0	1	1	1	1	6
Total for each item		11	3	9	6	1	6	8	3	11	11	69

**Table 2:** Characteristics of the studies' participants and interventions.

Study (year published) [ref.]	Participants	Age (intervention, control, or placebo group)	Modality	Treatments	Evaluation
Ardic et al. (2007) [16]	24 women with FM	43.5 ± 10.2, 48.8 ± 8.9	*Balneotherapy* 30°C, 20 min	Once daily, 5 days/wk for 3 wks, whole body; control	Pain (VAS), NTP, algometric score, FIQ, BDI, serum PGE2, LTB4, and IL-1 levels

Armagan et al. (2006) [17]	32 women with FM	38.9 ± 4.9, 37.6 ± 5.9	*Low-level laser therapy* 50 mW, 830 nm, 1 min each tender point	Once daily, 5 days/wk for 10 days; control	NTP, morning stiffness, VSGI, FIQ, and total myalgia score

Bagdatli et al. (2015) [18]	70 women with FM	45.2 ± 9.1, 42.8 ± 9.6	*Balneotherapy and mudpack* 38°C, 20 min and 45°C, 20 min	10 times within 2 wks, whole body; control	PGASc, IGASc, FIQ, pain, fatigue, sleep, stiffness, anxiety, depression, and BDI

Evcik et al. (2002) [19]	42 patients with FM	42.0 ± 6.8, 41.5 ± 7.1	*Balneotherapy* 36°C, 20 min	Once daily, 5 days/wk for 3 wks, whole body; control	Pain (VAS), FIQ, NTP, and BDI

Fioravanti et al. (2007) [20]	80 patients with FM	46.2 ± 10.5, 48.6 ± 9.4	*Mudpack and thermal bath* 40°C–45°C, 10 min and 37°C–38°C, 15 min	Once daily, for 2 wks, whole body; control	FIQ, VAS (headache, fatigue, sleep disturbances), NTP, HAQ, and AIMS

Gür et al. (2002) [21]	50 patients with FM	30.4 ± 6.9, 28.5 ± 6.3	*Low-level laser therapy* 2 J/cm^2^, 3 min each tender point	Once daily, 5 days/wk for 2 wks; placebo	Pain, NTP, skinfold tenderness, stiffness, sleep disturbance, muscle spasm, fatigue, and FIQ

Gür et al. (2002) [22]	40 patients with FM	—	*Low-level laser therapy* 11.2 mW, 3 min each tender point	Once daily, 5 days/wk for 2 wks; placebo	Pain, NTP, skinfold tenderness, stiffness, sleep disturbance, muscular spasm, and fatigue

Lauretti et al. (2013) [23]	39 patients with FM	32 ± 8, 35 ± 8	*TENS* 200 *μ*sec, 2 and 100 Hz, 60 mA, 20 min	Twice a day, for 7 days; placebo	Pain (VAS), daily analgesic consumption, quality of sleep, and fatigue

Ruaro et al. (2014) [24]	20 women with FM	43.4, 39.4	*Low-level laser therapy* 20 mW, 670 nm,7 s ×4 for 18 trigger points	3 times/wk for 4 wks; placebo	NTP, FIQ, McGill pain questionnaire, and VAS

Sutbeyaz et al. (2009) [25]	56 women with FM	43.0 ± 9.6, 40.9 ± 6.9	*Pulsed electromagnetic field therapy* 40 *µ*T, 0.1–64 Hz, 30 min	Twice a day, for 3 wks, whole body; control	FIQ, pain (VAS), BDI, SF-36, and PGART

Vayvay et al. (2016) [26]	45 women with FM	36.4 ± 8.3, 38.0 ± 8.4	*Laser therapy* 2 J/cm^2^, 3 min each trigger points	Once daily, 5 days/wk for 3 wks; placebo	Pain (VAS), body flexibility, FIQ, SF-36, and BDI

VAS: visual analogue scale; NTP: no. of tender points; BDI: Beck's depression index; FIQ: Fibromyalgia Impact Questionnaire; PGE2: prostaglandin E2; LTB4: leukotriene B4; IL: interleukin; VSGI: global improvement as reported on a verbal scale; PGASc: patient's global assessment score; IGASc: investigator's global assessment score; HAQ: health assessment questionnaire; AIMS: arthritis impact measurement scale; HDRS: Hamilton depression rate scale; DSM: diagnostic and statistical manual of mental disorders; SF-36: 36-item short form health survey; PGART: patient's global assessment of response to therapy.
